# Progress of research and application of *Heyndrickxia coagulans* (*Bacillus coagulans*) as probiotic bacteria

**DOI:** 10.3389/fcimb.2024.1415790

**Published:** 2024-05-28

**Authors:** Jie Liang, Chunhai Li, Zouquan Chen, Fangyu Guo, Jiaxin Dou, Ting Wang, Zhen Shang Xu

**Affiliations:** ^1^State Key Laboratory of Biobased Material and Green Papermaking, Qilu University of Technology, Shandong Academy of Science, Jinan, China; ^2^School of Bioengineering, Qilu University of Technology, Shandong Academy of Science, Jinan, China; ^3^Qilu Hospital, Shandong University, Jinan, Shandong, China

**Keywords:** probiotics, *Heyndrickxia coagulans*, safety assessment, secondary metabolism, probiotic functions

## Abstract

Probiotics are defined as living or dead bacteria and their byproducts that maintain the balance of the intestinal microbiome. They are non-toxic, non-pathogenic, and do not release any toxins either within or outside the body. Adequate consumption of probiotics can enhance metabolite production, increase immunity, maintain a balanced intestinal flora, and stimulate growth. Probiotics do not have negative antibiotic effects and help maintain the natural flora in animals in a balanced state or prevent dysbacteriosis. *Heyndrickxia coagulans* (*H. coagulans*) is a novel probiotic species that is gradually being used for the improvement of human health. Compared to commonly used probiotic lactic acid bacteria, *H. coagulans* can produce spores, which provide the species with high resistance to adverse conditions. Even though they are transient residents of the gut, beneficial bacteria can have a significant impact on the microbiota because they can outnumber harmful germs, and vice versa. This article discusses the probiotic mechanisms of *H. coagulans* and outlines the requirements for a substance to be classified as a probiotic. It also addresses how to assess strains that have recently been discovered to possess probiotic properties.

## 1 Background of the study

Whether alive or dead, probiotic microorganisms exist. Their metabolites keep the digestive tract’s microecological ecosystem in balance, which can enhance the ratio of beneficial bacteria there, inhibit harmful bacteria, boost the body’s immunity, and stimulate growth once the host consumes enough of them (Sun et al., 2022). Some of them can generate advantageous metabolites. They are non-toxic, non-pathogenic, free of side effects, and do not carry genes that could lead to the spread of antibiotic resistance. They do not contain any endogenous or exogenous toxins. They aid in maintaining the equilibrium of the animal’s normal bacterial flora or guard against bacterial malfunctions. Unlike antibiotics, they have antibacterial activity but none of the negative side effects (Wang et al., 2019). They take up space in the intestinal environment and exude antibiotic substances that prevent the growth of other microbes that could also produce antimicrobial effects. Due to their ability to digest complex carbohydrates and produce short-chain fatty acids like lactic acid and butyric acid, they can effectively utilize their probiotic supplements (Ong et al., 2016). Butyrate improves tightly knit tissues, reduces bacterial translocation, and encourages the production of mucin. This glycoprotein preserves the integrity of the intestinal epithelium and generates bacteriostatic substances that prevent the spread of infections (Sasaki et al., 2020). These bacteriocins exhibit a range of inhibitory effects, with studies indicating that certain probiotic strains can effectively suppress a variety of bacterial pathogens, as well as specific viruses and fungi. These bacteriocins exhibit a range of inhibitory effects, with studies indicating that certain probiotic strains can effectively suppress a variety of bacterial pathogens, as well as specific viruses and fungi. This broad-spectrum antagonistic activity is a critical aspect of their role in modulating the microbiota and in the prevention and treatment of various infectious diseases. Additionally, they face competition from other microbes for scarce nutrients (Yadav et al., 2022). Iron is a limited resource because it is a component of practically all living things. Mucus production, chloride and water secretion, and tight junctions that include the apical region of epithelial cells all contribute to the maintenance of intestinal barrier function. Some probiotics generate hydrolytic enzymes that decrease the pH of the intestines and raise the levels of short-chain fatty acids like butyric, propionic, and lactic acids (Keller et al., 2019). Maintaining a low pH results in a physiologically constrictive environment that prevents harmful microorganisms from growing and colonizing. Improvement of Mucosal Barrier Integrity Probiotics’ capacity to stick to the intestinal epithelium and mucus allows them to compete with infections and stop pathogen invasion through the epithelium. This mechanism prevents pathogen adherence to the intestinal system’s mucosa and epithelium.

*H. coagulans* is a Gram-positive, facultatively anaerobic, and neutral bacterium, classified as a lactic acid-forming species within the Bacillus genus (Mu and Cong, 2019). Its cells are cylindrical, with blunt ends, and can be found as single cells, in pairs, or occasionally in short chains. These bacteria produce spores at the tips and do not have flagella. Optimal conditions for growth include a temperature range of 30–50°C and a pH level of 5.5–6.5 (Chen et al., 2020). *H. coagulans* can ferment various substrates such as maltodextrin, mannitol, raffinose, sucrose, and trehalose, resulting in the production of acid without gas. The distinctive feature of *H. coagulans* is the location of its oval endospores, which are situated at the bacterial pole, contrasting with the central or subterminal positioning in other Bacillus species (Lee et al., 2016). The median genome size for this bacterium is 3.42411 Mb (megabase pairs), with a median count of 3,190 proteins and a median guanine-cytosine (GC) content of 46.5%. It is differentiated from other bacilli by the absence of cytochrome c oxidase and the inability to reduce nitrate to nitrite. Different strains of *H. coagulans* require varying growth factors, and while the specific requirements may differ with temperature, biotin and thiocyanin are essential for growth in most strains, as they cannot synthesize these themselves and must obtain them from external sources (Lee et al., 2016). The spores of *H. coagulans* are highly resistant to gastric acid, enabling them to reach the small intestine and germinate within the host’s gastrointestinal tract. The typical germination time is 4–6 hours after ingestion. However, due to their weak adhesion to intestinal epithelial cells, these bacteria generally have a transient presence in the intestinal tract, with the majority being eliminated through defecation within 4 to 7 days after ingestion (Peng et al., 2020). To maximize the probiotic effects of *H. coagulans*, it must be consumed regularly, as it shares health-promoting characteristics with Lactobacillus bifidobacterium, such as balancing the gut microbiota, aiding in nutrient metabolism, and enhancing immunity (Juturu and Wu, 2016). The spores of *H. coagulans* exhibit high-stress resistance, including the ability to withstand high temperatures, acidic conditions, and bile salts. This allows them to survive in environments where common probiotics cannot, and then germinate and reproduce at the right time, offering a significant advantage over other probiotics (Hiramoto et al., 2023). This feature also overcomes the limitations of common probiotics that are sensitive to environmental conditions. In aquaculture, *H. coagulans* is often used as an alternative to antibiotics. In the medical field, it is utilized in the production of live bacterial tablets and serves as an additive in the food industry and health care products. Its application is based on the use of the bacteria or its metabolites to regulate the body’s physical and chemical properties, thereby promoting the growth of beneficial bacteria and inhibiting harmful ones, which in turn improves the body’s immune system (Chang et al., 2021). Additionally, in a genomic study, the taxonomic status of Bacillus acidicola, Bacillus pervagus, and members of the genera Heyndrickxia, Margalitia, and Weizmannia was re-evaluated. The study used 16S rRNA gene sequence similarity, amino acid identity (AAI) values, and phylogenomic analysis to support the classification of Bacillus acidicola together with members of the genera Heyndrickxia, Margalitia, and Weizmannia into the same genus. Meanwhile, Bacillus pervagus was differentiated and proposed to be reclassified into a newly established genus, Oikeobacillus (Narsing Rao et al., 2023).

While *H. coagulans* serves as a beneficial probiotic, it also has certain limitations in its application. The bacterium exhibits weak adhesion to intestinal epithelial cells, which prevents long-term colonization and typically results in clearance through defecation within 4–7 days (Abhari et al., 2020). Continuous supplementation is required to maintain its probiotic effects, which could impact patient compliance. Moreover, its growth is contingent on specific temperature and humidity conditions, and it has particular requirements for growth factors such as biotin and thiamine. The absence of enzymes like cytochrome C oxidase restricts its application in certain biological processes. Genomic characteristics also influence its stability and functionality (Tarik et al., 2022). Although its highly resilient spores can withstand high temperatures, acidity, and bile salts, they require appropriate conditions to be activated, which may limit its application in mild environments. The probiotic effects can vary among individuals and are influenced by dosage and frequency. As a “transient” in the gut, its capacity to regulate the ecological balance of the microbiota is also limited. Therefore, when utilizing *H. coagulans* as a probiotic, these limitations must be considered, and further research and development are necessary to optimize its application (Huang et al., 2023).

## 2 Probiotic *Heyndrickxia coagulans*: genomic insights and secondary metabolite potential

### 2.1 Application of integrated molecular techniques in strain identification

**Figure 1:**
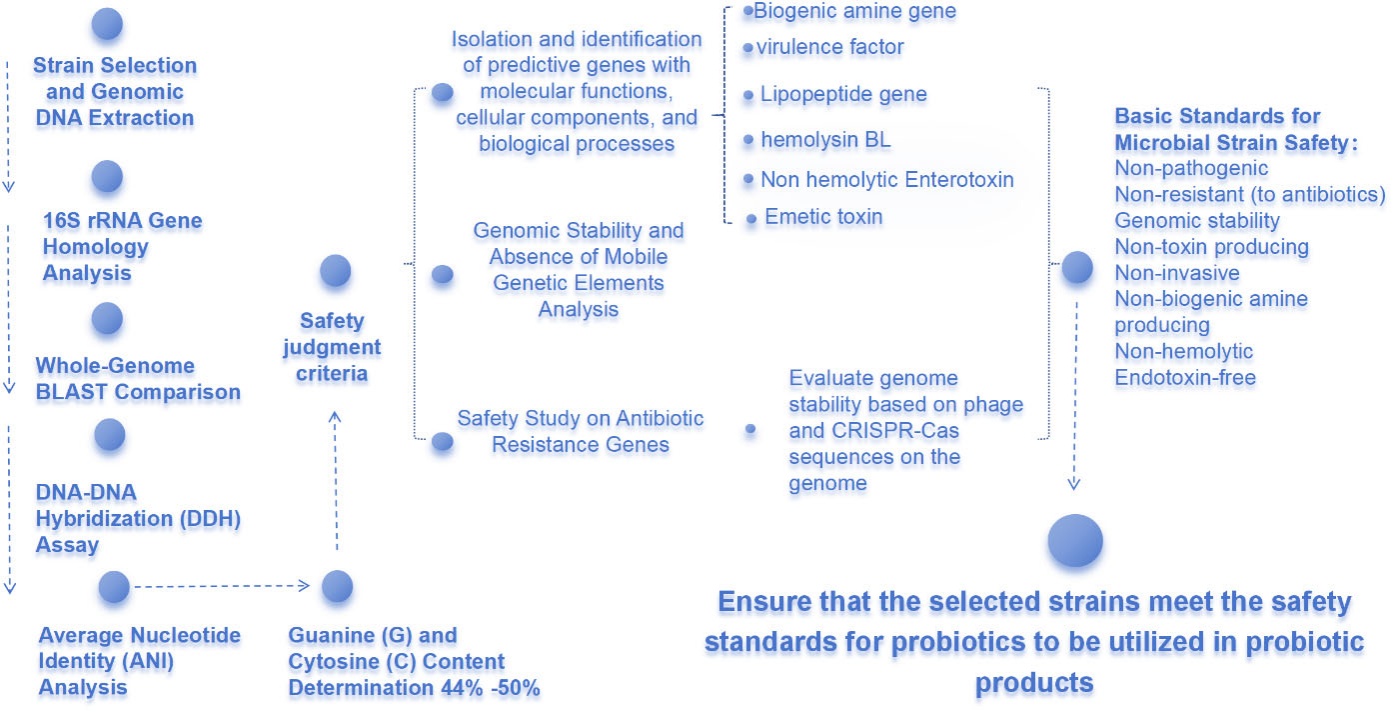
Flow Chart of Safety Identification of *Heyndrickxia coagulans*.

To ensure that *H. coagulans* meets the criteria for a safe probiotic strain, a suite of molecular biological techniques has been employed. This includes 16S rRNA gene homology analysis, whole-genome BLAST comparison, DNA-DNA hybridization (DDH) assays, and determination of the guanine (G) and cytosine (C) base ratio (G+C content). These methods are crucial for establishing the taxonomic affiliation of the strain. According to internationally recognized classifications, the genus Bacillus encompasses 77 species with a G+C content varying between 44% and 50% (Li, Liu et al., 2022). The quantitative correlation between DDH values and genome-derived parameters, such as Average Nucleotide Identity (ANI), provides a significant basis for strain classification at the species level (**Figure 1**).

### 2.2 Safety assessment of *Heyndrickxia coagulans* from a genomic perspective

In the realm of probiotics, the safety assessment of *H. coagulans* is a critical step prior to commercialization. This article delves into the genomic sequence of *H. coagulans* to conduct an in-depth analysis of its safety, with a focus on potential risk-related sequences within its genome, including antibiotic resistance genes, biogenic amine-producing genes, and virulence factor genes (Anaya-Loyola et al., 2019).

### 2.3 Genomic stability and absence of mobile genetic elements

*H. coagulans* strains, classified as spore-forming bacteria and recognized as Generally Recognized As Safe (GRAS), exhibit a notable absence of mobile genetic elements such as transposons and intact prophage sequences. This absence significantly reduces the risk of genetic information transfer, ensuring a non-pathogenic phenotype (Kang et al., 2021). The discovery of the CRISPR-Cas system adds an additional layer of protection for genomic stability, limiting the invasion of foreign DNA. Furthermore, the Restriction-Modification (R-M) system, in conjunction with the CRISPR-Cas system, enhances the strain’s defense mechanisms against bacteriophages and exogenous genetic elements (Zhao et al., 2021).

### 2.4 Safety studies on antibiotic resistance genes

The spread of antibiotic resistance is a significant challenge to global public health. Genomic sequence analysis of *H. coagulans* reveals the presence of a few genes encoding for antibiotic resistance; however, the absence of mobile elements such as transposons around these genes indicates a lack of transferable resistance (Konuray and Erginkaya, 2018). This finding confirms the low risk of *H. coagulans* in the dissemination of antibiotic resistance, aligning with strict safety standards for antibiotics.

### 2.5 Safety considerations for biogenic amine and virulence factor genes

The accumulation of biogenic amines can lead to toxic effects. However, the biogenic amine genes in *H. coagulans* are typically non-functional or expressed at very low levels, insufficient for the synthesis of significant amounts of biogenic amines. Additionally, the virulence factor genes, lipopeptide genes, and toxin-encoding genes in *H. coagulans* exhibit low homology with known toxic genes, and the integrity analysis of the genome shows a lack of complete phage sequences, further ensuring genomic stability (Konuray and Erginkaya, 2018). Collective analysis indicates that *H. coagulans* does not exhibit pathogenicity to humans or animals, thus validating its safety for probiotic applications.

### 2.6 Biological characteristics of *Heyndrickxia coagulans*

#### 2.6.1 Stress resistance and growth characteristics

**Figure 2:**
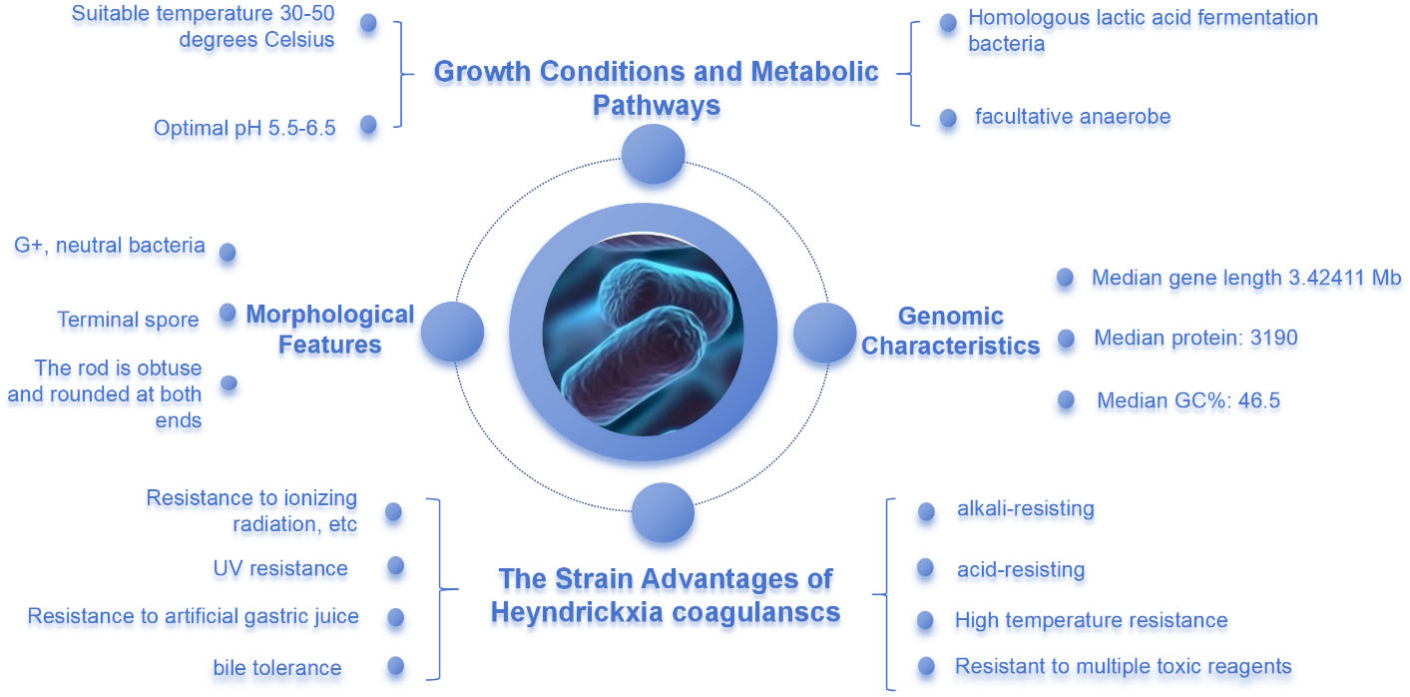
Biological Characteristics of *Heyndrickxia coagulans*.

*H. coagulans* can withstand extreme conditions such as ultraviolet radiation, ionizing radiation, high temperatures, desiccation, and various toxic solvents. Coupled with a high tolerance to acidity and bile, these traits set *H. coagulans* apart in the probiotic field. The spore state of *H. coagulans* allows it to survive adverse environments and germinate at the right time, a feat not commonly achieved by general probiotics (**Figure 2**).

#### 2.6.2 Inhibitory properties

The inhibitory capabilities of *H. coagulans* are primarily realized through two modes: “competitive exclusion” and “colonization resistance.” In the modulation of the gut microbiota, *H. coagulans* interacts with the microbial community through metabolic antagonism, mutualism, and the production of antimicrobial substances (Mu and Cong, 2019). The production of bacteriocins and pediocins during its growth not only directly inhibits the growth of pathogenic bacteria but also participates in the regulation of the gut environment as quorum-sensing molecules (Sun et al., 2022).

#### 2.6.3 Promotion of beneficial bacteria

*H. coagulans* exerts its probiotic effects by secreting metabolic products and synergizing with other microbial communities. Its facultative anaerobic nature allows it to form spores under specific conditions and rapidly germinate into vegetative cells in the gastrointestinal tract, thereby promoting the growth and colonization of beneficial bacteria such as *Lactobacillus* and *Bifidobacterium*. Moreover, the main metabolic product of *H. coagulans*, L-lactic acid, lowers the gastrointestinal pH, creating a more favorable environment for anaerobic probiotics (Abdhul et al., 2015). This process not only optimizes the structure of the microbial community but also enhances the host’s absorption and utilization of inorganic trace elements through the formation of organic mineral salts. *H. coagulans* also secretes specialized adhesion proteins, including mucins and fibrinogen-binding proteins, which facilitate colonization in the gut and reduce pathogenic adhesion (Zhao et al., 2023).

### 2.7 Genomic sequence analysis and secondary metabolite biosynthetic gene clusters of *Heyndrickxia coagulans*

In this article, we have selected known genomic sequences of *H. coagulans* and conducted an in-depth analysis using the AntiSMASH tool. The aim is to identify and characterize potential secondary metabolite biosynthetic gene clusters (BGCs) within *H. coagulans*, which is crucial for understanding its functionality in probiotic applications. The data analysis presented in the table reveals a diversity of BGCs within the *H. coagulans* genome, which may be responsible for synthesizing secondary metabolites with antibacterial, antioxidant, or other biological activities (**Table 1**).

**Table 1:** Heyndrickxia coagulans Strain-Specific Secondary Metabolite Biosynthetic Gene Clusters Identified by AntiSMASH.

Strain	Antismash-type(size)	Most similar known cluster(Similarity)
*H. coagulans ASRS217*	1.T3PKS(215185–256357) 2.RiPP-like(2659400–2669738) 3.Betalactone(2892294–2920089) 4.RiPP-like(3393893–34064120)	2.Amylocyclicin(50%) 3.Fengycin(33%)
*H. coagulans BC01*	T3PKS(226977–268149) Thiopeptide(1073808–1098825) RiPP-like(2672264–2682602) Betalactone(2914924–2943305) Terpene(2956623–2978488) RiPP-like(3426725–3436952)	3. Amylocyclicin(50%) 4. Fengycin (40%) 5.Legonindolizidine(8%)
*H. coagulans DSM 2314*	1.RiPP-like(323230–333568) 2.Betalactone(529699–557495) 3.RiPP-like(1018620–1028847) 4.LAP(1238486–1261580) 5.T3PKS(1413480)	1.Amylocyclicin(50%) 2.Fengycin (33%) 4.listeriolysin S (37%)
*H. coagulans FDAARGOS_1160*	T3PKS(1573717–1614889) Epipeptide(2191893–2213615)	–
*H. coagulans HOM5301*	T3PKS(217822–258994) Thiopeptide,LAP(1107940–1132955) RiPP-like(2713700–2724038) Betalactone(2929010–2956802)	3.Amylocyclicin(50%) 4.Fengycin (40%)
*H. coagulans IDCC1201*	RiPP-like(1947678–1958016) Betalactone(2124507–2153007) RiPP-like(2645063–2655290) T3PKS(3073385–3114569)	1.Amylocyclicin(50%) 2.Fengycin (40%)
*H. coagulans JBI-YZ6.3*	T3PKS(225228–266412) RiPP-like(2692626–2702964) Betalactone(2879246–2907064) Terpene(2909196–2931061) Epipeptide(3182649–3204371) RiPP-like(3371682–3381909)	1.Amylocyclicin(50%) 2.Fengycin (33%) 3.Legonindolizidine(8%)
*H. coagulans LA204*	LAP(89293–112397) T3PKS(266003–307187) RiPP-like(2817380–2827718) Betalactone(3021109–3048905) RiPP-like(3511998–3522225)	1. listeriolysin S (37%) 3.Amylocyclicin(50%) 4.Fengycin (33%)
*H. coagulans S-lac*	RiPP-like(244719–255057) Betalactone(421549–450049) RiPP-like(943737–953964) T3PKS(1368873–1410057)	1.Fengycin (40%)
*H. coagulans terpene*	Thiopeptide,LAP(362914–387931) RiPP-like(1964777–1975115) Betalactone(2194844–2223225) Terpene(2236543–2258408) RiPP-like(2706642–2716869) T3PKS(3115159–3156331)	2.Amylocyclicin(50%) 3.Fengycin (33%) 4.Legonindolizidine(8%)
*H. coagulans ATCC 7050*	Epipeptide(1853126–1874848) T3PKS(2513409–2554581)	2.locillomycin/locillomycin B/locillomycin C
*H. coagulans VHProbi C08*	1.T3PKS(274569–315753) 2.RiPP-like(2820698–2831036) 3.Betalactone(2997528–3026028) 4.RiPP-like(3519649–3529876)	3.Fengycin (40%)

Natural products with diverse structures and rich biological activities, including Type III Polyketide Synthases (T3PKS), RiPP-like molecules, β-lactones (Betalactone), thiopeptides, lantipeptides (LAP), and terpenes (Terpene), are widely distributed in bacteria, plants, and fungi. These compounds not only demonstrate the diversity of biosynthetic pathways but also reflect their important roles in biological defense mechanisms (Dolati et al., 2023). T3PKS catalyzes the synthesis of polyketide compounds with antibacterial, anti-inflammatory, and antitumor effects in bacteria and plants. RiPP-like molecules, after ribosomal synthesis, undergo special modifications such as cyclization and oxidation to form compounds with antibacterial, antiviral, and antifungal activities. β-Lactones, with their sulfur-containing four-membered ring structures, are commonly found in certain antibiotics and exhibit significant biological activity. Thiopeptides, a class of natural products produced by bacteria, are known for their sulfur rings and mixed peptide bonds, demonstrating potent antibacterial, antifungal, and antitumor activities. LAPs, containing non-standard amino acids such as lanthionine and dehydroalanine, are generated through special enzymatic modification reactions and possess a variety of biological activities (Chaudhari et al., 2022). Terpenes, an important component of plant secondary metabolites, composed of isoprene units, have multiple biological activities, including anti-inflammatory, antioxidant, antimicrobial, and pest control, as well as immune system regulation.

The discovery and research of these natural products not only enrich our understanding of biosynthetic pathways but also provide valuable candidate molecules for new drug development. With the advancement of synthetic biology and genomics technologies, there will be more opportunities in the future to engineer these complex molecules to enhance their biological activity and stability, thereby playing a greater role in the fields of medicine, agriculture, and industrial biotechnology.

## 3 Probiotic properties and mechanism of action of *Heyndrickxia coagulans*

**Figure 3:**
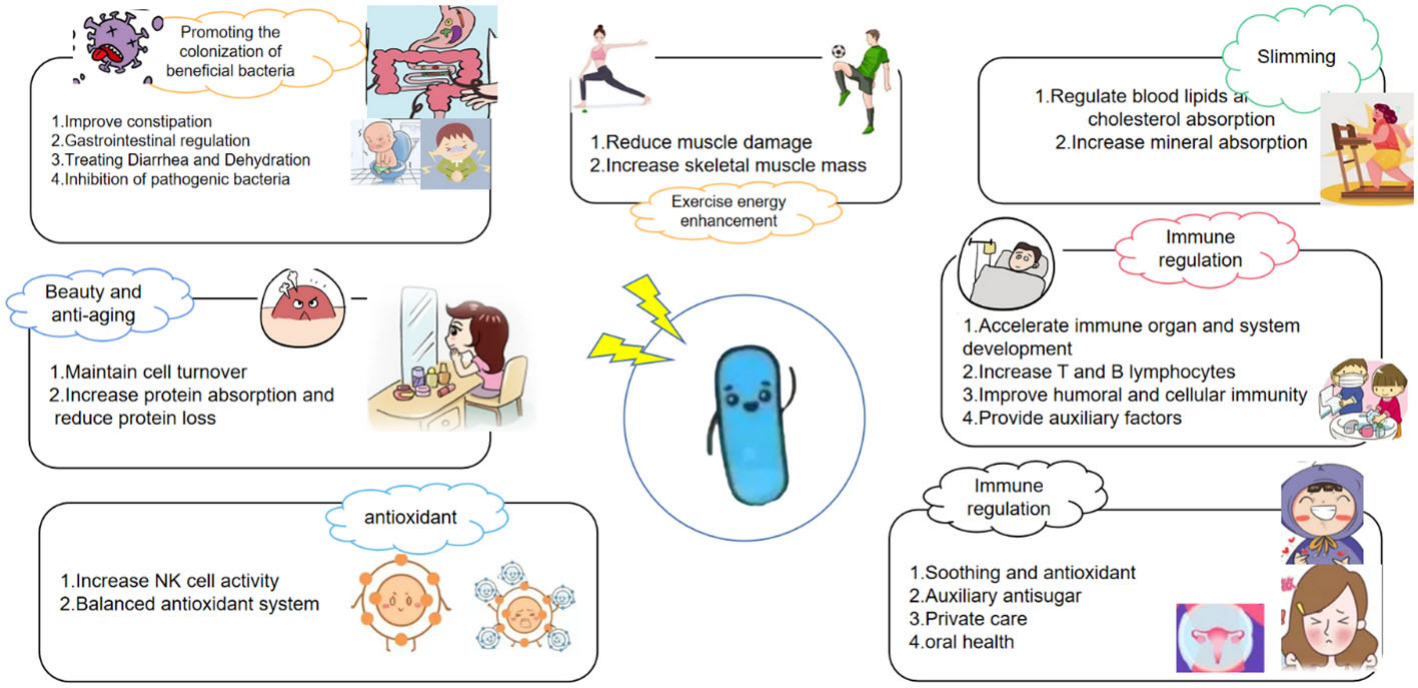
Prebiotic characteristics of *Heyndrickxia coagulans*.

**Figure 4:**
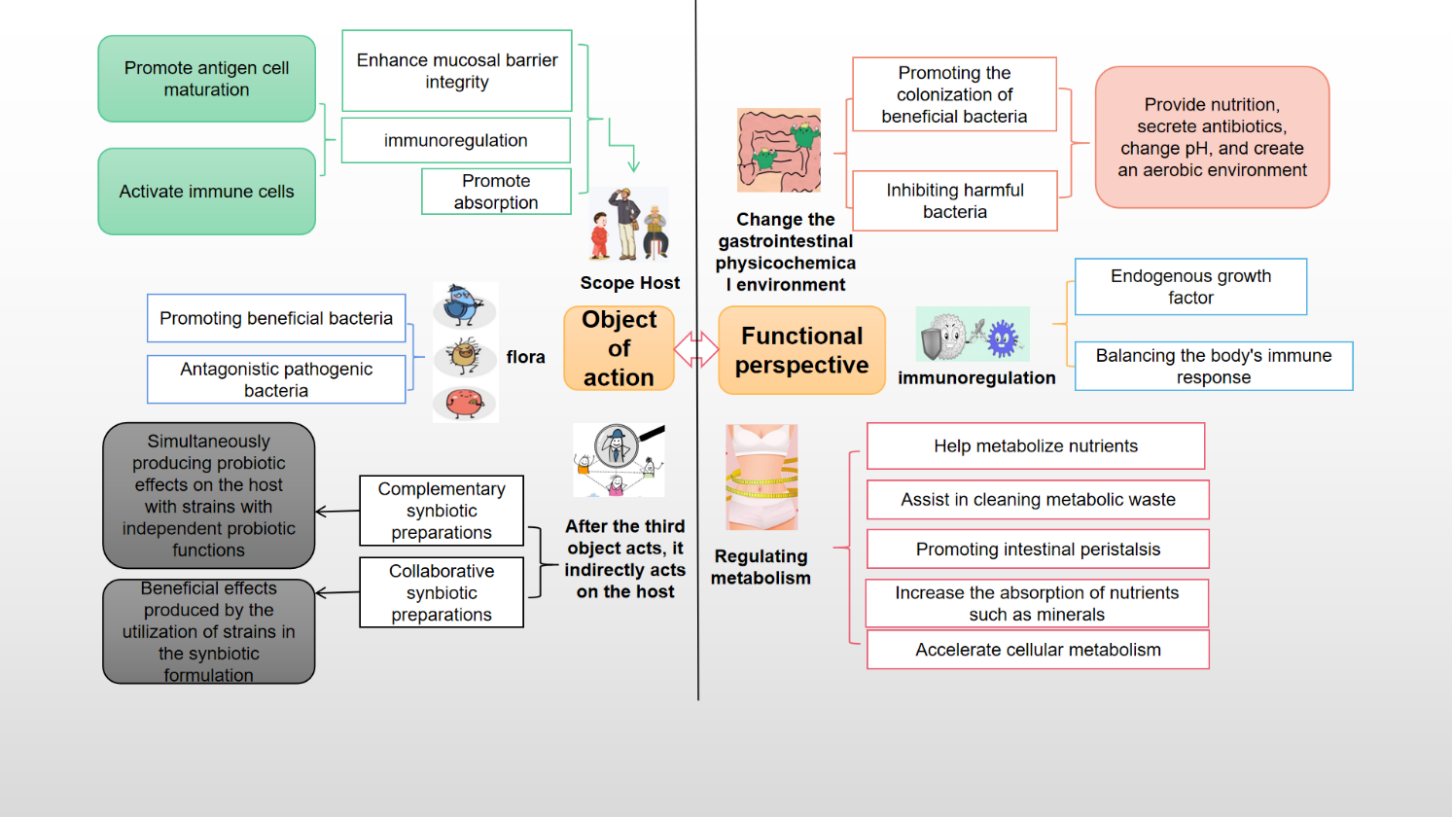
The probiotic mechanism of *Heyndrickxia coagulans* was analyzed from the point of view of target and function.

*H. coagulans*, a probiotic, offers a multitude of benefits, encompassing gastrointestinal health, exercise performance, weight management, immune modulation, systemic well-being, and disease prevention. It also contributes to beauty and anti-aging, counters oxidative stress, and enhances skincare. Additionally, *H. coagulans* has demonstrated potential in cancer prevention and possesses antipruritic and antiallergenic properties (Guo et al., 2021). The mechanisms underlying its probiotic effects vary and are detailed separately according to their specific targets and outcomes (**Figures 3**, **4**).

### 3.1 Gastrointestinal conditioning to assist in the fight against pylorus

The human digestive system is initially sterile at birth but quickly gets populated by trillions of bacteria and hundreds of different species of microorganisms. The colon contains the highest microbial content, and the density and diversity of bacteria increase exponentially from the stomach to this organ (Abdhul et al., 2015). The human gut microbiota is dominated by thick-walled bacteria and the phylum Mycobacterium, which also contains a core group of microbes with related roles. The immune system and intestinal epithelium must develop and mature in the presence of symbiotic microbes (Batra et al., 2011). Through mechanisms of colonization resistance and fermentation of indigestible carbohydrates, which mostly take place un the proximal colon, the gut microbiota protects against pathogens. In addition to having anti-inflammatory and antioxidant potential, butyrate influences intestinal epithelial cell proliferation, differentiation, mucus secretion, and barrier function. Direct antibacterial action, improved mucosal barrier integrity, and immunomodulation are the more precise ways by which it modifies the gut flora and produces flora metabolites (Saroj and Gupta, 2020). Antibacterial peptides, secreted as part of the bacteriocin complex during the growth phase, are capable of penetrating the cell surface of pathogenic bacteria. This disruption leads to the leakage of intracellular amino acids and inorganic salts, inducing metabolic disturbances within the pathogenic bacteria. It will also secrete a variety of digestive enzymes, such as lipase, protease, amylase, -galactosidase, -galactosidase, xylanase, and others, which can speed up the intestinal tract’s metabolic rate by breaking down large molecules like proteins and starch, and help the animal digest the nutrients in the feed (Zhai et al., 2021).

### 3.2 Exercise boosts energy, slims the body and reduat, enhances endurance

For persons who exercise for extended periods of time, *H. coagulans* may lessen the muscle damage brought on by exercise and improve skeletal muscle mass. It is well known that mTOR, a mechanistic protein kinase target of rapamycin, is an important regulator of MPS and muscle mass. Protein consumption following weight-bearing exercise increases the effects of both dietary activation of mTOR and mechanical stimulation of MPS (Liu et al., 2015). Rapidly digested proteins include a high concentration of essential amino acids (EAA), the crucial amino acid of which appears to be leucine, which activates MPS more potently than other proteins. Consuming more protein does not further increase MPS. Leucine absorption is enhanced by *H. coagulans* and certain probiotics, which boost EAA absorption. Adherence to a full-body workout routine four times per week can lessen muscle damage, accelerate the rate of response to training, and lessen exercise-induced muscle injury when combined with a strength training program. It can lessen the amount of fat and cholesterol that is absorbed in the intestines, which aids in the favorable regulation of blood lipids. It can speed up the digestion of food in the digestive tract, increase the synthesis of vitamins B and K as well as other healthy compounds, which is effective in boosting the body’s metabolism and enhancing digestion. It can also accelerate the absorption of calcium, magnesium, and other minerals. Additionally, it helps lower body fat, cleanses intestinal waste, and prevents fat accumulation, all of which have a substantial positive impact on figure control (Gao et al., 2023).

### 3.3 Immunomodulation, systemic conditioning, and disease prevention

*H. coagulans* increases the number of T- and B-lymphocytes, phagocytosis, and secretion by mono- and macro-phagocytes, as well as the level of humoral and cellular immunity, the effectiveness of which is primarily dependent on the nucleus accumbens. It also effectively recognizes antigenic sites in the digestive tract with immune-adjuvant function and puts the relevant lymphoid tissues in a state of intense immune readiness (Su et al., 2011). Since the intestinal epithelial cells’ adherence to bacteria makes it impossible for them to survive in the intestinal tract naturally, some dead bacteria can supply some nutrients, while other probiotics can help because of this. In contrast, when a certain level of *H. coagulans* is reached in the intestinal tract, compounds that encourage the synthesis of probiotic bacteria can be generated, making the probiotic bacteria dominant. Phosphomimetic acid, lysophosphatidic acid (LTA), exocytosis polysaccharides, S-layer-associated protein (SLAP), mucin-binding protein (MUB), fibronectin-binding egg, and bacterial hyphae are all present on its cell surface structures. Many probiotics share these proteins or non-protein macromolecules, which offer interoperable probiotic processes and functionalities. Binding to host intestinal epithelial cells, a mucus layer, or immune cells that recognize or control one another serves to illustrate this (Bomko et al., 2017).

*H. coagulans* can be used to treat injuries caused by the alkylating agent cyclophosphamide (CTX)-induced damage by modulating the intestinal origin and the fecal microbiota. For example, *H. coagulans* (BCS) can be used in combination with the prebiotic fructooligosaccharides (FOS) to treat injuries caused by CTX-induced damage. An alkylating drug called cyclophosphamide is largely employed in the treatment of cancer. With its great immunosuppressive efficacy, CTX is being used more frequently to treat autoimmune and hereditary immunological disorders. CTX promotes a Th1/Th2 shift in the cytokine profile, which forms the molecular basis of its anti-metastatic effects. However, a dysbiosis of the microbiota may result from this translocation (Hoffman et al., 2019). The maintenance of the mucosal barrier and the Th1/Th2 balance is aided by CTX’s capacity to lower serum immunoglobulin levels and raise them via BCS and BCS + FOS.

### 3.4 Beauty and anti-aging, anti-oxidation, beauty, and skincare

*H. coagulans*, a species of Gram-positive bacteria, has lipopolysaccharide (LPS) as a significant component of its outer membrane. LPS acts as a potent activator of the innate immune response, leading to the production of both pro-inflammatory and anti-inflammatory mediators, as well as reactive oxygen species (ROS). If an excess of free radicals accumulates in cells and tissues without being sufficiently neutralized by antioxidants, the body’s homeostasis can be disrupted. Oxidative damage to cells and tissues may occur when the stress surpasses a certain threshold (Wauters et al., 2021). To maintain the structural and functional integrity of cell membranes, two essential enzymes, glutathione peroxidase (GSH-Px) and superoxide dismutase (SOD), neutralize toxic intracellular compounds and halt peroxidation reactions. Malondialdehyde (MDA), a byproduct of lipid oxidation, indicates the extent of membrane system damage through its concentration in the body. Catalase (CAT), a key enzyme protein, scavenges peroxidative agents and protects the body from injury following oxidative or phagocytic processes. By enhancing the activity of GSH-Px, SOD, and CAT, and reducing MDA levels, *H. coagulans* can mitigate oxidative stress. Furthermore, it can stimulate the body’s production of endogenous growth factors, which is advantageous for cellular metabolism, accelerates the clearance of metabolic waste, eliminates senescent cells, enhances the body’s resistance, and bolsters immunity, ensuring that cells remain vital, and promoting skin cell renewal for a more youthful and effective skin state (Hashemnia et al., 2023). The extracellular polysaccharides produced by *H. coagulans* possess antioxidant properties, can inhibit pathogenic bacteria, and help maintain the digestive tract’s microecological balance. They also boost the activity of natural killer (NK) cells and both humoral and cellular immunity in animals. However, the beneficial bacteria, when consumed, are often gradually lost through fecal elimination, necessitating regular consumption to sustain functionality. A sudden surge in oxidative stress can lead to oxidative damage and potentially trigger a cascade of inflammatory responses. The body’s immune system relies heavily on a robust antioxidant system for its integrity (Aulitto et al., 2021). *H. coagulans* is capable of metabolizing and producing essential nutrients such as niacin, pantothenic acid, biotin, vitamin B6, vitamin B12, and folic acid, which are vital cofactors for the body’s metabolic processes and physiological function and can be supplemented as vitamins.

### 3.5 Other probiotic attributes

*H. coagulans*, a Gram-positive bacterium, contains lipopolysaccharide (LPS) in its outer membrane, which acts as a potent stimulator of the innate immune system. This activation can lead to the production of both pro-inflammatory and anti-inflammatory cytokines, as well as reactive oxygen species (ROS), which are involved in various immune responses (Liu et al., 2022). *H. coagulans* has been shown to support immune regulation and potentially alleviate allergy symptoms by modulating cytokine expression and enhancing phagocytosis. It may also improve the bioavailability of amino acids from plant proteins in the small intestine, thus aiding in the absorption of these nutrients and reducing the risk of their fermentation into toxic metabolites by the gut microbiota. The strains of *H. coagulans* are non-mutagenic, non-teratogenic, and non-genotoxic, which is essential for their safety in consumption. Furthermore, *H. coagulans* can help maintain a balanced immune response, potentially reducing the severity of allergic reactions and the incidence of skin conditions such as eczema (Jung et al., 2022). It also plays a role in preserving the delicate flora balance, particularly in the reproductive system, where it can prevent the overgrowth of pathogenic bacteria by maintaining an acidic environment, thus reducing the risk of recurrent infections often associated with antibiotic use.

## 4 Application status

### 4.1 Clinical applications

**Figure 5:**
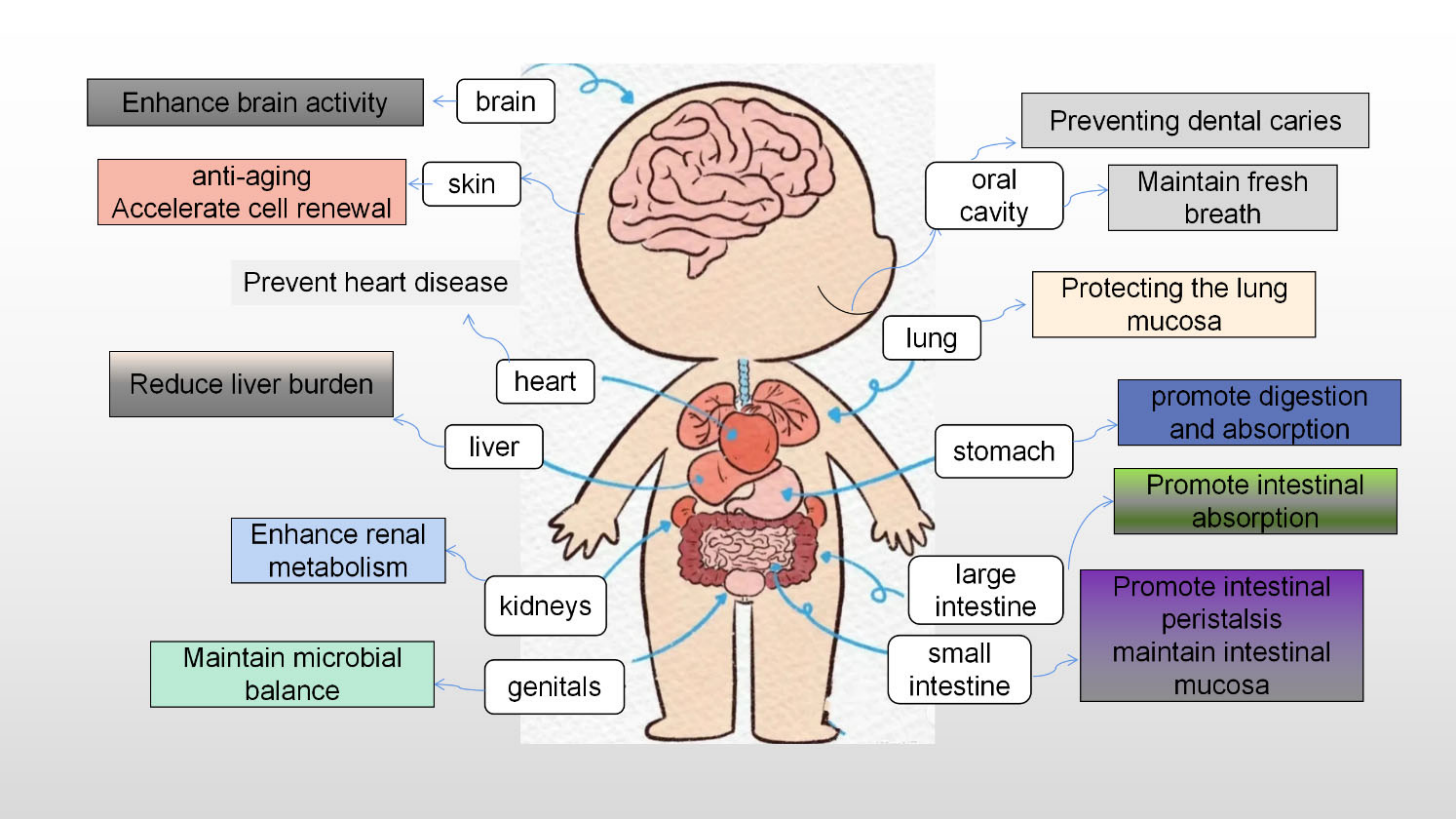
The effects of *Heyndrickxia coagulans* on different organs of the human body.

The beneficial effects of probiotics include altering the composition and activity of the microbiota, improving epithelial barrier performance, altering immune and systemic metabolic responses, and signaling through the central nervous system that various body systems are capable of producing additional probiotic effects. *Lactobacillus*, *Bifidobacterium*, *Enterococcus*, *Streptococcus*, *Bacillus*, *Saccharomyces*, *Propionibacterium*, *Streptococcus gastric*, *Pediococcus*, *Anaplasma*, and *Akkermansia* are some of the most diverse probiotics that have emerged in recent years. *H. coagulans* is one of these species that produces endophytes, and some strains, known as *Lactobacillus sporogenes* or *Bacillus sporogenes*, have a variety of advantageous effects, such as modulating metabolism, immunological response, and microbiota composition (**Figure 5**). A promising pharmaceutical preparation for the regulation of gastrointestinal disorders, dental caries, vaginitis, rheumatoid arthritis, heavy metal toxicity, exercise, and hyperlipidemia is one that has high stability in production, storage, transportation, and administration and is better suited for use in food and pharmaceutical formulations (Urtasun et al., 2020).

### 4.2 Intestinal microecology

The intestinal barrier, which guards against harmful substances like bacteria and toxins from invading the body’s other tissues, organs, and blood circulation, is made up of the intestinal mucosal epithelium, intestinal mucus, intestinal flora, secretory immunoglobulins, and intestinal-associated lymphoid tissue. When the intestinal barrier is broken, there is an increase in intestinal permeability, pathogens enter the body and cause systemic inflammation, and high free radical concentrations in the intestines can be cytotoxic to the phospholipids in the membranes of the intestinal epithelial cells (Khatri et al., 2016). After being effectively treated with *H. coagulans*, the spores are activated in the stomach’s acidic environment, where they germinate and multiply, generating an acidic environment that is detrimental to inflammatory bacteria (Gao et al., 2022). The main cause of inflammatory bowel injury is oxidative stress, which can harm DNA, proteins, and lipids when the body’s amount of oxygen free radicals rises. Both an increase in reactive oxygen species (ROS) and lipid peroxidation can harm the gut. The simultaneous breakdown of macromolecules yields a wide range of metabolites; secretion of some of the active compounds can be in the intestinal tract to create a biological barrier of protection, to encourage mucosal immune response, and secretion of bacteriocins on the intestinal microorganisms have broad-spectrum activity, not only to affect the colonization of the intestinal bacterial flora but also to improve the body’s immune system.

#### 4.2.1 Regulation of intestinal metabolism and microenvironment

A strain of the genus Bacillus that can produce lactic acid is called *H. coagulans*. It can lower the pH of the surrounding environment, which inhibits the growth of some pathogens and aids other intestinal bacteria in the fermentation of short-chain fatty acids, regulating intestinal metabolism and the microenvironment (Jäger et al., 2018). Through the induction of increased production of butyric acid, SCFA (primarily acetic acid, propionic acid, and butyric acid) have the potential to significantly improve the phenotype of cardiovascular metabolism, maintain healthy blood pressure, regulate the insulin response, and reduce the risk of type 2 diabetes mellitus and cardiovascular disease. Constipation is a common intestinal issue that affects both youngsters and the elderly. In comparison to mono-mineral-oil treatments, the combination of synthetic lactobacilli(R) composed of *H. coagulans* and mineral oil (paraffin) led to a significant increase in bowel movement frequency as well as a reduction in fecal straining and incomplete bowel movements (Li et al., 2022). AAD, or antibiotic-associated diarrhea, can result from improper antibiotic use. By lessening the harm that antibiotics cause to the natural intestinal flora, *H. coagulans* can be used concurrently with antibiotic therapy as a therapeutic adjuvant. C. difficile is a bacterium that colonizes the intestines and can result in antibiotic-associated colitis, diarrhea, and even mortality in cases of Clostridium difficile-associated diarrhea (CDAD) (Chaudhari et al., 2022). Orally ingesting *H. coagulans* probiotic supplements can help reduce Clostridium difficile infections and their sequelae, especially when used in conjunction with antibiotics. Idiopathic inflammatory bowel illnesses of the intestinal tract, ulcerative colitis (UC) and Crohn’s disease (CD), both exhibit clinically as diarrhea, abdominal pain, and bloody stools. Supplementing with certain probiotics can improve ulcerative colitis but not Crohn’s disease, which is closely related to intestinal dysbiosis.

#### 4.2.2 Maintenance of intestinal tissues

By enhancing the integrity of the intestinal epithelial barrier and regulating mucus secretion, *H. coagulans* primarily acts to preserve the health of intestinal tissues. Damage to the intercellular tight junctions, bacterial membranes, and intestinal mucosal epithelial cells—components that serve as mechanical barriers against external interference—due to inflammation or stress indicates a compromised intestinal barrier. The intestinal tight junction structure is highly dynamic, and changes in permeability in response to external stimuli can significantly affect the lymphatic system, potentially leading to systemic inflammation, damage to tight junction proteins (referred to as “leaky gut”), diarrhea, inflammatory bowel disease, and other non-gastrointestinal conditions. *H. coagulans* promotes the maturation of goblet cells, which are unicellular glands located between the columnar epithelium of the small intestinal mucosa (Dolati et al., 2023). These cells secrete large amounts of mucin and contribute to the mucosal barrier. They help maintain the intestinal tract’s self-healing ability by increasing the villus-to-crypt depth ratio. Irritable bowel syndrome (IBS), a chronic gastrointestinal disorder, presents with symptoms such as abdominal bloating, pain, diarrhea, and/or constipation, which can markedly reduce the patient’s quality of life. *H. coagulans* can encourage intestinal peristalsis and improve bowel movements, potentially avoiding diarrhea and alleviating constipation, dehydration, and other symptoms. It helps maintain a balanced intestinal flora, suppresses the growth of harmful bacteria, and prevents the production of toxins that can irritate the intestinal mucosa and lead to diarrhea. Compared to a placebo group, treatment with *H. coagulans* and simethicone for IBS has been shown to significantly reduce pain intensity and improve fecal consistency, abdominal discomfort, bloating, staining, urgency, incomplete evacuation, and gas passage in the probiotic group (Su and Xu, 2014).

### 4.3 Protecting the stomach health

Due to its spore-producing abilities, *H. coagulans* can survive the harsh environment of the stomach acid, allowing it to reach the small intestine. It has been shown to improve human digestion by promoting the secretion of digestive enzymes and aiding in the absorption and utilization of proteins. By potentially modifying intestinal pH, *H. coagulans* may enhance mineral absorption and contribute to a gut environment that supports the production of essential compounds by the gut microbiota. Functional dyspepsia is a common gastrointestinal disorder characterized by symptoms such as bloating and discomfort in the upper abdomen (Yao et al., 2016). Despite there being no structural abnormalities in routine examinations, the condition can significantly impact quality of life, and current treatments may be expensive with variable safety profiles. Proton pump inhibitors (PPIs) are the first-line treatment for functional dyspepsia, but their long-term use can sometimes lead to gut microbiota dysbiosis. *H. coagulans* and other probiotics like Bacillus subtilis have shown potential as safe and effective alternatives or adjuncts to PPIs, particularly in individuals with functional dyspepsia who experience persistent symptoms. Small intestinal bacterial overgrowth (SIBO) is a condition that can arise from the use of PPIs and may be addressed by spore-forming probiotics such as *H. coagulans*, which could be considered as a monotherapy or an add-on to PPI treatment under medical guidance.

### 4.4 Stabilization of the macroecology of the oral, reproductive, and dermal systems

Additionally, Candida-related infections of the mucous membranes of the intestines, vagina, and oral cavity are treated and prevented using *H. coagulans*. The produced antimicrobials are also utilized to prevent uropathogens and treat related urinary system and reproductive tract infections by competitively limiting Candida colonization and growth and so lowering clinical symptoms. The most prevalent vaginal infection in the world, bacterial vaginosis is characterized by a reduction in lactobacilli in the vaginal environment. Treatment for this illness with antibiotics frequently fails. Women with vaginosis who have undergone antibiotic medication that disrupts the flora can be treated with *H. coagulans* (Majlesi et al., 2017). Women who have a history of recurrent bacterial or fungal vaginitis, urinary tract infections, or bacterial infections being treated with antibiotics may find it useful as a supplement to antibiotics. It can assist in restoring the body’s normal biological flora, but it does not lessen the risk of infection or stop the sickness from returning.

The balance of the skin flora represents the health of the skin and the symbiotic interaction between the microbial population and the host. The skin is the largest organ in the body. It is necessary for healthy skin to maintain a tightly managed and sensitive equilibrium. Atopic dermatitis (AD), psoriasis, and non-melanoma skin cancer are just a few of the skin disorders that have been linked to skin dysbiosis. Studies on wound healing or rapid wound healing have also shown the importance of probiotics in wound protection (Jäger et al., 2018). Lowering pH and maintaining interspecies competitive exclusion can stop harmful bacterial development in situations where there are open wounds.

### 4.5 Immunomodulation

*H. coagulans* administration drastically modifies mucosal immunity. Epithelial cells, dendritic cells (dc), T-cells, regulatory T-cells (Treg), monocytes/macrophages, immunoglobulin A (IgA)-producing B-cells, and natural killer cells are just a few of the host cell types it can affect. It can also cause T-cell apoptosis. The dc penetrates the epithelium and enters the intestinal lumen, where it can provide antigens crucial for early bacterial identification and the development of T-cell responses. It also has the ability to activate Treg cells, which explains how it has an anti-inflammatory effect and helps treat a number of inflammatory disorders, such as Crohn’s disease and atopic dermatiti. Immune responses may also be regulated by the creation of B-lymphocytes and antibodies against potentially damaging antigens (Khatri et al., 2016). Numerous active antigens found on the surface of the bacteria can trigger the body’s non-specific immune response, boost the activity of natural killer cells and phagocytes in the intestinal villi, and increase IgA secretion. *H. coagulans* and Lactobacillus plantarum, when taken orally, reduced the amount of mercury in the liver and kidneys and prevented alterations in glutathione peroxidase and superoxide dismutase levels, which helped the host fight mercury intoxication. Cadmium is a hazardous heavy metal that causes oxidative stress in people. By reducing the buildup of the enzymes aspartate aminotransferase (AST), alanine aminotransferase (ALT), total bilirubin, blood urea nitrogen, and metals in the liver and kidneys, as well as by elevating sodium dismutase levels in the liver and serum, co-inulin may be resistant to the toxic effects of heavy metals (Gao et al., 2023).

## 5 Summary outlook

Future genetic research will further clarify the molecular mechanisms behind the advantageous benefits of *H. coagulans* as more genomic data on this organism becomes available. The most typical use is the creation of live *H. coagulans* tablets, which when used for the clinical efficacy in the treatment of infant eczema have been shown to reduce the rate of recurrence and shorten the time to regression of lesions and disappearance of syndromes. Patients with alcoholic fatty liver disease, pediatric diarrhea, and hand, foot, and mouth disease benefit from its safe therapeutic effects in addition to treating juvenile eczema. It provides great relief for common intestinal disorders such as constipation, chronic colitis, pediatric pneumonia, and diarrhea. It is safe and may be supplemented with drugs that treat rheumatoid arthritis. Regular use can reduce the duration of respiratory infections in children and prevent gastrointestinal issues like diarrhea. Live bacterial preparations with *H. coagulans* can also be used to treat symptoms of irritable bowel syndrome. A chronic inflammatory condition known as ulcerative colitis (UC) is brought on by intestinal barrier dysfunction and intestinal microbiota dysbiosis, both of which affect the immune system. In addition to helping to reduce inflammation, *H. coagulans* and prebiotics can effectively treat ulcerative colitis. Due to its beneficial industrial and probiotic qualities, *H. coagulans* is identified as a promising probiotic for treating intestinal illnesses. Infantile colic in neonatal diarrhea has good efficacy in preventing neonates and premature infants with clinical problems such as necrotizing enterocolitis of the small intestine (NEC). By using *H. coagulans* as a cell factory to produce galactooligosaccharides (GOS), which can improve food digestion and nutrient absorption, it is very effective to deliver these enzymes to the stomach. As a result, *H. coagulans*, a newly discovered probiotic, has remarkable probiotic properties.

## Author contributions

JL: Writing – original draft, Writing – review & editing, Conceptualization, Data curation, Formal Analysis, Funding acquisition, Investigation, Methodology, Project administration, Resources, Software, Supervision, Validation, Visualization. CL: Writing – original draft, Conceptualization, Data curation, Formal Analysis, Funding acquisition, Investigation, Methodology, Project administration, Resources, Software, Supervision, Validation, Visualization. ZC: Investigation, Writing – review & editing. FG: Conceptualization, Methodology, Writing – review & editing. JD: Formal Analysis, Validation, Writing – review & editing. TW: Formal Analysis, Funding acquisition, Visualization, Writing – original draft, Writing – review & editing. ZX: Project administration, Supervision, Writing – review & editing, Writing – original draft.
